# Silent Nucleotide Polymorphisms and a Phylogeny for *Mycobacterium tuberculosis*

**DOI:** 10.3201/eid1009.040046

**Published:** 2004-09

**Authors:** Lucy Baker, Tim Brown, Martin C. Maiden, Francis Drobniewski

**Affiliations:** * Health Protection Agency, London, United Kingdom;; † University of Oxford, Oxford, United Kingdom

**Keywords:** Molecular evolution, bacterial genetics, Mycobacterium tuberculosis, Mycobacterium bovis, phylogeny, polymorphism, tuberculosis

## Abstract

Much remains unknown of the phylogeny and evolution of *Mycobacterium tuberculosis*, an organism that kills 2 million people annually. Using a population-based approach that analyzes multiple loci around the chromosome, we demonstrate that neutral genetic variation in genes associated with antimicrobial drug resistance has sufficient variation to construct a robust phylogenetic tree for *M. tuberculosis*. The data describe a clonal population with a minimum of four distinct *M. tuberculosis* lineages, closely related to *M. bovis*. The lineages are strongly geographically associated. Nucleotide substitutions proven to cause drug resistance are distributed throughout the tree, whereas nonsynonymous base substitutions unrelated to drug resistance have a restricted distribution. The phylogenetic structure is concordant with all the previously described genotypic and phenotypic groupings of *M. tuberculosis* strains and provides a unifying framework for both epidemiologic and evolutionary analysis of *M. tuberculosis* populations.

## Abstract 2

Population diversity of genetically silent nucleotide polymorphisms produces a unifying phylogeny for *Mycobacterium tuberculosis*.

*Mycobacterium tuberculosis* has caused tuberculosis (TB) in humans for thousands of years (*1**,**2*), and the World Health Organization (WHO) estimates that one third of the global population is infected with *M. tuberculosis* (*3*); however, the bacterium has remained an enigma. The global resurgence of TB highlights the need for an improved understanding of its epidemiology and its evolutionary biologic features. Recent advances in molecular characterization of *M. tuberculosis* isolates, which index variation in insertion sequences (*4*) and repetitive genomic elements (*5**,**6*), have elucidated clusters of identical and closely related strain families (*7**–**9*). These findings have provided insights into regional (*10*) and national (*11*) epidemiologic features. However, these techniques may be less suited to global population and evolutionary analyses, and integrating information obtained from different approaches is often complex (*12*). Genomic comparisons have identified genetic variation for population screening; however, these analyses are limited to those sites that vary between the compared genomes and are potentially misleading (*13**–**15*). Nucleotide sequences provide robust, portable, and comparable data for studying population variation. The mutational processes that generate this variation are understood, and sequence data have been successfully used in the study of bacterial epidemiology, population biology, and evolution (*16*). The complete genome sequences (*15**–**18*) provide access to all regions of the chromosome and facilitate such studies. However, high-throughput gene sequencing of structural genes (*19*) and host immune system protein targets (*20*) in *M. tuberculosis* isolates indicated low levels of sequence diversity. Although extensive genomic sequencing was performed in both studies, comparable sequence data were obtained on a limited number of highly selected isolates.

We used an unbiased population approach to analyze genetically silent nucleotide sequence variation for seven unlinked loci distributed around the chromosome. The loci chosen were genes associated with antimicrobial drug resistance that have been reported to possess >95% of all sequence variation observed in 26 structural genes studied (*19*), which includes >90% of synonymous nucleotide substitutions, i.e., nucleotide substitutions that do not affect the translated amino acid. In a population sample of 316 U.K. clinical isolates, silent single nucleotide polymorphisms (sSNPs) resolve an unambiguous phylogeny and provide a unifying framework for epidemiologic, population, and evolutionary analyses.

## Methods

### Bacterial Strains

The 316 *M. tuberculosis* clinical isolates were identified in England and Wales from January 1, 1998, through December 31, 1998, and included all the viable clinical isolates (n = 216) resistant to one or both of the firstline antituberculous drugs (rifampicin and isoniazid) and 100 randomly chosen fully susceptible isolates. One *M. tuberculosis* H37Rv isolate, four *M. bovis* isolates, two *M. africanum* type I isolates, and one *M. microti* isolate were included for comparison. *M. tuberculosis* complex isolates were identified with a combination of microscopic and macroscopic appearance, growth characteristics, biochemical analysis, and DNA hybridization (*21*). Clinical and epidemiologic information were obtained from laboratory records at the HPA Mycobacterium Reference Unit, London, UK, the U.K. Mycobacterial Resistance Network database (MYCOBNET), and the 1998 national TB survey (*22*).Duplicate isolates were excluded. Drug susceptibility was determined by the resistance ratio method (*2*). Strains were characterized by IS*6110* restriction fragment length polymorphism (RFLP) and spoligotyping (*4**,**5*)

### Amplifying and Sequencing Target Gene Loci

The nucleotide sequences were obtained for the following seven gene loci: *rpoB*, *katG*, *oxyR*, *ahpC*, *pncA*, *rpsL*, and *gyrA*. These gene loci are associated with drug resistance, but without antimicrobial drug selection pressure, they would be regarded as housekeeping genes. Primers and amplification conditions for polymerase chain reaction (PCR) are shown in Table 1. Products were purified by precipitation with 20% polyethylene glycol–2.5 mol/L NaCl and sequenced from both DNA strands by using internal nested primers (Table 2) and BigDye Ready Reaction Mix (ABI, Warrington, UK) according to the manufacturer's instructions. Unincorporated dye terminators were removed by precipitation with 96% ethanol–0.115 mol/L sodium acetate, pH 4.6. The reaction products were separated and detected with an ABI prism 3700.

**Table 1:** Amplification primers

Gene/locus	Forward primer	Reverse primer	Product (bp)	Reaction conditions	Cycles
gyrA	gyrA-ext F 5´-ACAGACACGACGTTGCCGCC-3´	gyrA-ext R 5´-GTCGATTTCCCTCAGCATCTCC-3´	435	95°C for 15 min 95°C for 15 s 68°C for 30 s 72°C for 1 min 72°C for 5 min	30^b^
inhA	mabA-ext F 5´-TCGTAGGGCGTCAATACACC-3´	mabA-ext R 5´-TCATTCGACCGAATTTGTTG-3´	605	94°C for 5 min 94°C for 30 s 60°C for 30 s 72°C for 30 s 72°C for 5 min	30
katG	katG-ext3F 5´-CGACGAAATGGGACAACAGT-3´	katG-ext3R 5´-TGCATGAGCATTATCCCGTA-3´	1,507	94°C for 5 min 94°C for 30 s 60°C for 30 s 72°C for 1 min 72°C for 7 min	30
	katG -ext5F 5´-TCGACTGTGCTGTTGGCGAGG-3´	katG-ext5R 5´-CTTCGCCGACGAGGTCGTGG-3´	1,531	95°C for 15 min 95°C for 30 s 68°C for 30 s 72°C for 1 min 72°C for 7 min	30^b^
oxyR-ahpC	oxyR-ext F 5´-TCGAGCTGCGACGGTGCTGG-3´	oxyR-extR 5´-CTGCGGGTGATTGAGCTCAGG-3´	1,437	95°C for 15 min 95°C for 30 s 72°C for 30 s 72°C for 1 min 72°C for 7 min	30^b^
pncA	pncA-ext F 5´-AACCAAGGACTTCCACATCG-3´	pncA-extAR 5´-CAGAAACTGCAGCATCATCG-3´	1,324	95°C for 15 min 95°C for 30 s 64°C for 30 s 72°C for 1 min 72°C for 7 min	35^b^
rpoB	rpoB-46F 5´-GGCCGTGGGCACCGCTCC-3´	rpoB 1868R 5´-CCAGCGGGGCCTCGCTACG-3´	1,822 bp	95°C for 15 min 95°C for 15 s 65°C for 30 s 72°C for 3 min 72°C for 10 min	30^b^
	rpoB 1711F 5´-GTGCCCTCGTCTGAGGTGGAC-3´	rpoB 3602R 5´-AAGACCGATGCGGAGTTCATCG-3´	1,891	95°C for 15 min 95°C for 15 s 65°C for 30 s 72°C for 3 min 72°C for 10 min	30^b^
rpsL	rpsL-extF 5´-GGCCGACAAACAGAACGT-3´	rpsL-extR 5´-GTTCACCAACTGGGTGAC-3´	494	94°C for 5 min 94°C for 30 s 56°C for 30 s 72°C for 30 s 72°C for 5 min	30

^a^A final PCR reaction volume of 25 µL was used that contained 2.5 µL of 10 x ammonium sulfate reaction buffer (Bioline, London, UK), 1.5 mmol/L magnesium chloride (Bioline); 200 µmol/L each of dATP, dTTP, dGTP, and dCTP; 300 nmol/L of each primer pair, 0.8 units of Taq DNA polymerase (Bioline); 1 µL (≈10 ng) of template DNA and sterile distilled water. Amplification was carried out in 0.2-mL thin-wall polymerase chain reaction tubes in a DNA Thermal Cycler 9600 (Applied Biosystems, Warrington, UK). Products were purified by precipitation with 20% polyethylene detected with an ABI Prism 3700 or an ABI Prism 377 automated DNA sequencer (ABI, Warrington, UK) . ^b^Reaction performed with Hotstar Taq and reaction buffer (Qiagen, Crawley, UK).

**Table 2:** Sequencing primers^a^

Locus/PCR product	Forward primers	Reverse primers
*gyrA*	gyrA-1F 5´-CAGCTACATCGACTATG-3´	gyrA-1R 5´-GGGCTTCGGTGTACCTCAT-3´
*inhA* promoter	mabA-1F 5´-AGAAAGGGATCCGTCATGGT-3´	mabA-1R 5´-GTCACATTCGACGCCAAAC-3´
*katG 3F-3R*	katG-1F 5´-ACGCGGGGTCTGACAAAT-3´	katG-1R 5´-GACAAGGCGAACCTGCTTAC-3´
	katg-2F 5´-GTAAGCAGGTTCGCCTTGT-3´	katG-2R 5´-TCGGGATTGACTGTCTCACA-3´
	katG-3F 5´-ATCTCTTCCAGGGTGCGAAT-3´	katG-3R 5´-GAGTGGGAGCTGACGAAGAG-3´
*katG 5F-5R*	katG-4F 5´-AGAGGTCAGTGGCCAGCAT-3´	katG-4R 5´-AGATGGGGCTGATCTACGTG-3´
	katG-5F 5´-GCTGTTTCGACGTCGTTCAT-3´	katG-5R 5´-ACTACGGGCCGCTGTTTATC-3´
	katG-6R 5´-ACACTTCGCGATCACATCC-3´	katG-6R 5´-ACACTTCGCGATCACATCC-3´
*oxyR-ahpC*	oxyR–1 5´-CTGGCCAGGTAAGACGACC-3´	oxyR-2 5´-CAGACGCTCGATGCTGCC-3´
	oxyR-7 5´-TCATATCGAGAATGCTTGCGG-3´	oxyR-4 5´-TGCTTGGCGTCCACCTTGG-3´
	oxyR-6 5´-TGATGTCTTTGGCGTACTCGG–3´	oxyR-6 5´-CAATGACGAGTTCGAGGACC-3´
*pncA*	pncA-P1 5´-GCTGGTCATGTTCGCGATCG-3´	pncA-R 5´-CGATGAAGGTGTCGTAGAAGC-3´
	pncA –F 5´-AACCAAGGACTTCCACATCG–3´	pncA-2F 5´-ATACCGACCACATCGACCTC-3´
*rpoB–46-1868*	rpoB –41F 5´-GTGGGCACCGCTCCTCTAAGG-3´	rpoB 509R 5´-TGACCACCACACGCTCGGTCC-3´
	rpoB 331F 5´-CGTTTCGACGATGTCAAGGCA-3´	rpoB 975R 5´-GTCGACGACGTGATGGGCTCG-3´
	rpoB 783F 5´-CTGGAGAAGGACAACACCGTCG-3´	rpoB 2 5´-GCACGTCGCGGACCTCCAGCC-3´
	rpoB 1 5´-GGTCGGCATGTCGCGGATGGA-3´	rpoB 1845R 5´-CGCTACGGACCAGCGGCACC-3´
*rpoB 1711-3602*	rpoB 1725F 5´-GGTGGACTACATGGACGTCTC-3´	rpoB 2313R 5´-GTCGGAGATGTTCGGGATGTCG-3´
	rpoB 2134F 5´-GAGATGGCGCTGGGCAAGAAC-3´	rpoB 2770R 5´-TCTGGCCGATGTTCATCCGTCG-3´
	rpoB 2600F 5´-AGCTGGTGCGTGTGTATGTGG-3´	rpoB 3213R 5´-GGCCTGCATGCCCCAGCACTCC-3´
	rpoB 3013F 5´-CCGTTCCCGTACCCGGTCACG-3´	rpoB 3581R 5´-GAAGAAGTTGACGTCGAGCAC-3´
*rpsL*	rpsL F 5´-ACGTGAAAGCGCCCAAGATAGA -3´	rpsL R 5´-ACCAACTGCGATCCGTAGACC-3´

^a^All sequencing reactions were performed in 96-well plates (Abgene, UK) in a DNA Thermal Cycler 9600 (Applied Biosystems, Warrington, UK) by using the following thermocycling conditions: 30 cycles of denaturation at 96°C for 10 s, annealing at 50°C for 5 s, and extension at 60°C for 2 min.

### Genetic Analysis

Sequences were assembled with the STADEN suite of computer programs (*23*). The sequences were compared, and isolates with identical sequences were assigned the same allele number. For each gene, the DNA sequence was translated in frame, and each nucleotide polymorphism characterizing the allele was classified as synonymous or genetically silent, nonsynonymous, or intergenic.

For each isolate, the concatenated sequences from the coding region of all seven gene loci were reduced to a 36-nt sequence motif, constituting a synonymous sequence profile (SSP), and distinct SSPs were assigned a synonymous sequence type (SST). Phylogenetic analysis of the SST motifs was performed with the MEGA (*24*) and PHYLIP software packages (*25*): 225 isolates were sequenced at all loci. The sequencing data from the initial 225 isolates demonstrated key polymorphisms that were lineage defining. The remaining 94 isolates were assigned a lineage based on polymorphisms at *katG*^87^, *katG*^609^, *katG*^1388^, *oxyR*^37^, *oxyR*^285^, and *ahpC*^–46^, and by the spoligotype deletion pattern. A lineage was only ascribed if all data points agreed (Table 3). The relationship between lineages, phenotypic and genotypic drug resistance, and country of birth was analyzed with chi-square and Fisher exact test.

**Table 3:** Characteristic features of the four major lineages of *Mycobacterium tuberculosis* with respect to *M. bovis*

Species/lineage	TbD1 region of difference	Silent single nucleotide polymorphism						Nonsynonymous base substitutions		
		*rpoB* 3243 T	*rpoB* 2646 G	*katG* 87 A	*katG* 609 T	*oxyR* 285 A	*oxyR* 37 T	*katG* 1388 G	*ahpC* –46 A	Spoligotype signature spacer deletion
*M. tuberculosis* Lineage I	–	–	–	–	–	–	–	–	–	1–34
*M. tuberculosis* Lineage II	–	+	–	–	–	–	–	+	–	33–36
*M. tuberculosis* Lineage III	–	–	+	–	–	–	–	–	+	4–8, 23–24
*M. tuberculosis* Lineage IV	+	–	–	–	–	–	+	–	–	29–32 and 34
*M. bovis*	+	–	–	+	+	+	–	–	–	39–43

### Comparison with Outgroups

An in silico analysis of the seven gene loci was undertaken for two mycobacterial outgroups, *M. leprae* (*26*) and *M. marinum* with BLAST (Sanger Institute, Cambridge, UK; available from http://www.sanger.ac.uk/projects/M_marinum/). The complete gene sequences for each of the seven loci in *M. tuberculosis*, *M. bovis*, *M. leprae*, and *M. marinum* were aligned in frame by using Clustal-W. Two approaches were used. First, the aligned sequences for the coding regions of the seven gene loci were concatenated to produce a single sequence of 8.212 Kbp for each isolate. The concatenated sequences for fully susceptible examples of the *M. tuberculosis* SSTs were aligned to this. Second, SSPs were constructed for *M. leprae* and *M. marinum* by using the relevant aligned nucleotide for each of the previously identified variable synonymous sites in *M. tuberculosis* and *M. bovis*. For each approach, a phylogeny was constructed with the neighbor-joining tree method, and the results were compared.

### TbD1-PCR Analysis

Three isolates per SST were selected for analysis. Each possessed, when possible, a different IS*6110* RFLP or spoligotype pattern. This analysis was performed with the published method (*13*).

## Results

### Observed Genetic Diversity

The complete gene was sequenced for all but one locus, which provided 8,318 Kbp of nucleotide sequence data for each isolate. Across the seven loci, 115 variable sites were identified, of which 101 were within the coding region of the selected loci, and 36 were associated with genetically neutral base substitutions. The number of alleles per locus varied from 6 (*oxyR*) to 40 (*rpoB*). The proportion of variable sites present at each locus was low, 0.68% (*rpoB*) to 2.68% (*pncA*). Nonsynonymous base substitutions were more frequent than synonymous substitutions at almost all loci. The ratio of nonsynonymous substitutions per nonsynonymous site to synonymous substitutions per synonymous site (*d_N_/d_S_* ratio) varied from 0.109 to 0.848 in sensitive *M. tuberculosis* isolates and from 0.301 to 1.952 overall, which implied that resistance to antituberculous medication is indeed the selective force at most loci. Five variable sites were unique to *M. bovis*, of which four were associated with synonymous polymorphisms. A further variable site with a previously reported synonymous polymorphism (*katG* ^C609T^) (*27*) was identified in both *M. bovis* and *M. microti*, present in all four *M. bovis* isolates sequenced and the published *M. bovis* genome sequence (*18*).

### Synonymous Sequence Types and Lineages

**Figure 1:**
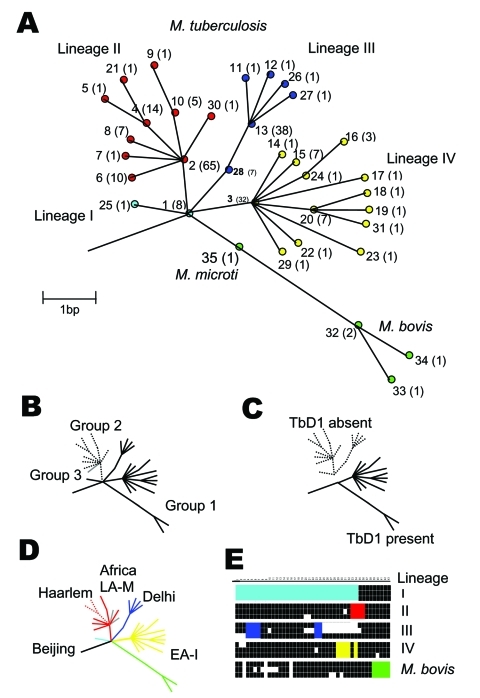
Unifying phylogeny for *Mycobacterium tuberculosis*. A) Maximum parsimony tree of *M. tuberculosis* and *M. bovis* based on 37 silent single-nucleotide polymorphisms in 225 isolates. Synonymous sequence types (SST) are marked 1–35. The frequency of each SST is marked in parentheses. The nodes of the major lineages are highlighted: lineage I (cyan), lineage II (red), lineage III (blue), lineage IV (yellow), and *M. bovis* (green). The colors correspond to those in Figure 2. Note both *M. africanum* Type I isolates sequenced were SST 1. B) Schematic representation of the genetic groups 1, 2, and 3 defined by the *katG-gyrA* scheme. C) Schematic representation of the presence or absence of the tuberculosis specific region of difference, TbD1. D) Schematic representation of the strain families Beijing, Haarlem, Africa, Delhi, East Africa-India (EA-I), and Latin America-Mediterranean (LA-M), previously described by IS*6110* restriction fragment length polymorphism typing and spoligotyping, demonstrating concordance with the phylogenetic tree. E) Spoligotyping patterns for representative isolates of each lineage demonstrating lack of probe hybridization at spacers 1–34 in lineage I, 33–36 in lineage II, 4–7 and 23–24 in lineage III, 29–32 and 34 in lineage IV, and 39–43 in *M. bovis*.

**Figure 2:**
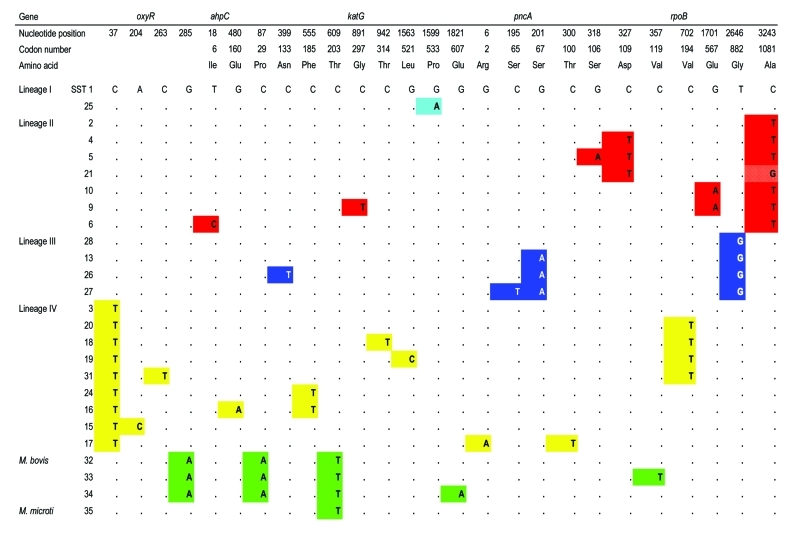
Relationship between the 37 silent single nucleotide polymorphisms, the synonymous sequence types, and major lineages.

By disregarding nonsynonymous polymorphisms, i.e., those producing an amino acid change likely in response to diversifying or stabilizing selection, a subset of 37 neutral sSNPs at 36 sites was generated; one site possessed two different synonymous substitutions. These substitutions occurred in 35 unique combinations, which we term synonymous sequence types (SSTs); each was assigned an arbitrary number (Figure 2). The variation in the SSTs conformed to the clonal model for bacterial population structure (*28*), and the maximum parsimony method generated a phylogenetic tree with no homoplasies, i.e., the lack of independent occurrence of a polymorphism in more than one branch of the tree (Figure 1A, Figure 2). Each branch corresponds to a unique combination of sSNPs. The phylogeny was robust, whether constructed with or without outgroup SSTs generated from the *M. leprae* and *M. marinum* genome sequences. The analysis identified four prominent *M. tuberculosis* lineages (numbered I to IV); the *M. bovis* isolates formed an additional lineage. The lineages are defined by distinct combinations of sSNPs (Figure 2). Virtually all of the nodes in the tree are occupied; internal nodes tend to be represented more frequently in the isolate population. Although a number of evolutionary scenarios are possible, the most likely explanation for this observation is that the sSNP variation arose recently.

Within the *M. tuberculosis* complex, the sSNPs clearly distinguish *M. tuberculosis* from *M. bovis* and *M. microti*, with the *M. microti* SST forming a node on the *M. bovis* lineage. The *M. africanum* type 1 isolates sequenced could not be distinguished from *M. tuberculosis* because they share SST-1.

### SST Phylogeny and Population Subdivisions

A variety of approaches have been used previously to subdivide *M. tuberculosis* strains into definable groups. These include assignments based on two nonsynonymous polymorphisms in *katG* and *gyrA* (*19*); the presence or absence of a TB-specific genomic region of difference, TbD1 (*13*); variation within the genomic direct repeat region demonstrated by spoligotyping; and strain families defined by highly conserved DNA fingerprint patterns obtained by RFLP of the insertion element IS*6110*. Each technique defines a limited number of distinct subdivisions, which although different, overlap when techniques are compared. The sSNP phylogenetic tree was congruent with all of the previously described subdivisions (Figure 1).

### *katG* and *gyrA* Polymorphisms

*M. tuberculosis* isolates can be divided into three groups (*1**–**3*) based on two apparently unselected nonsynonymous SNPs, *katG* ^G 1388 T^, and *gyrA*^G 284 C^ (*19*). Group 1 is defined by the combination of *katG* ^1388 T^ and *gyrA* ^284 C^, group 2 by *katG* ^1388 G^ and *gyrA* ^284 C^, and group 3 by *katG* ^1388 G^ and *gyrA* ^284 G^. We characterized the *katG*-*gyrA* polymorphisms in all isolates. The *katG* ^G 1388 T^ polymorphism cosegregated in all cases with the synonymous base substitution *rpoB* ^T 3243 C^ found in lineages I, III, IV, and *M. bovis*, whereas the *gyrA* ^G 284 C^ polymorphism subdivided SST-2 in lineage II. Group 1 isolates can therefore be subdivided into three prominent *M. tuberculosis* lineages (I, III, and IV) made up of 21 SSTs and *M. bovis*. Group 2 and 3 *M. tuberculosis* isolates are subdivisions of lineage II. Group 2 isolates can be further subdivided into 10 SSTs, whereas group 3 isolates are confined to a subdivision of SST 2 (Figure 1B). When the SST of the clinical isolate CDC1551 was identified in silico by analysis of the relevant sequences (www.tigr.org), it shared the same sequence type, SST 2, as the other isolate for which a complete genome is available, H37Rv. Although these two isolates are distinguished by numerous other synonymous and nonsynonymous polymorphisms (*15*), these organisms are closely related, which has implications for genetic variation based on comparing the two complete genome sequences (*14**,**29*).

### TbD1 Region of Difference

The presence or absence of DNA regions, identified by genomic comparisons of *M. tuberculosis* H37Rv and *M. bovis* BCG, can be used to distinguish the closely related members of the *M. tuberculosis* complex. However, only two groups of *M. tuberculosis* isolates have been described with this approach, defined by the presence or absence of the TB-specific region, TbD1 (*13*). TbD1 PCR analysis was performed on three epidemiologically unrelated isolates from each SST, when available. We found that the TbD1 region was present in all 13 SST types constituting lineage IV and all *M. bovis*, *M. microti*, and *M. africanum* type I isolates, but the region was absent from all other lineages and SSTs (Figure 1C). This finding implies that the TbD1 deletion occurred before both the *katG* ^G 1388 T^ and the *rpoB* ^T 3243 C^ mutations. Although SST-1 appears the least differentiated SST with the maximum parsimony method and sSNP data alone, when taken together with the TbD1 data, the ancestors of lineage IV are likely to have diverged from *M. africanum* type I, *M. microti*, and *M. bovis* before differentiation of the other *M. tuberculosis* lineages.

### DNA Fingerprinting Techniques

Molecular epidemiologic analyses of TB populations use a combination of typing techniques, most commonly IS*6110* RFLP analysis and spoligotyping. Spoligotyping has been used to distinguish members of the *M. tuberculosis* complex (*5**,**30*), and together with IS*6110* RFLP, has been used to describe various *M. tuberculosis* strain families including Beijing (*7*), Haarlem (*8*), Africa (*8*), Delhi (*9*), East Africa-India (EA-I), and Latin America-Mediterranean (*12*). Both techniques were used to type all 316 isolates, producing 234 IS*6110* RFLP patterns and 263 spoligotyping patterns; 157 isolates were assigned to strain families.

**Figure 3:**
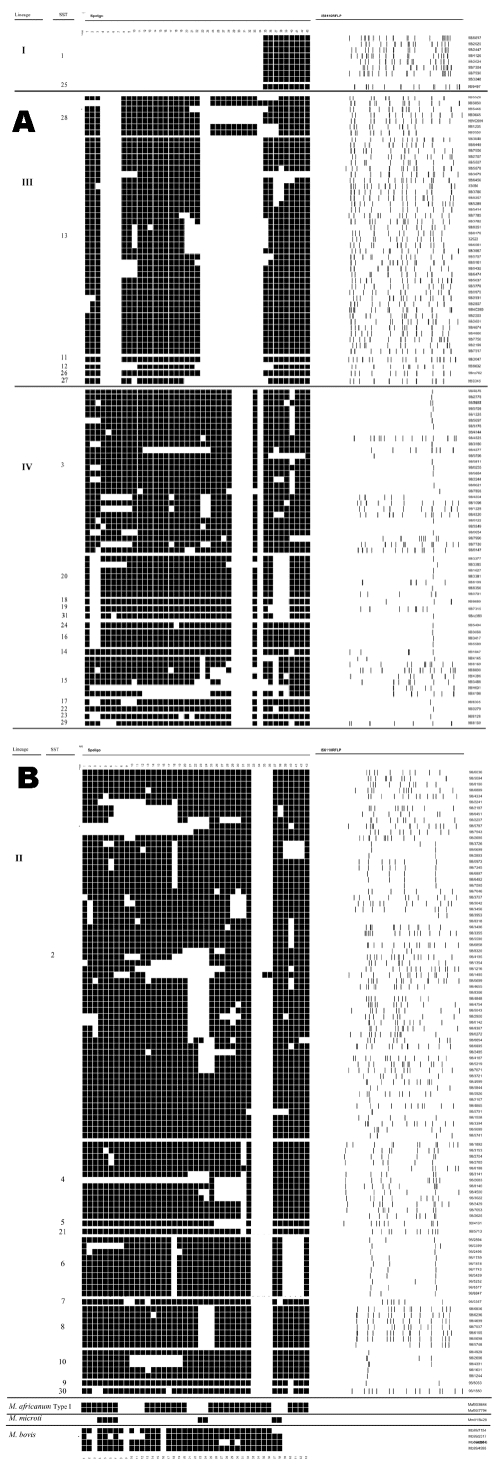
Relationship between *Mycobacterium tuberculosis* synonymous sequence type (SST), and lineage (left hand column), spoligotype pattern (middle column), and IS6110 restriction fragment length polymorphism.

All isolates within each family were confined to a single lineage (Figure 1D). Furthermore, each lineage was defined by a distinct pattern of spacer deletions (Figure 1E). The signature spoligotype spacer deletion pattern for lineage II (lack of probe hybridization at spacers 33–36) concurs with that previously noted in group 2 and group 3 *M. tuberculosis* isolates (*13**,**31*). Lineage I was defined by the signature spoligotype of the Beijing family (absence of spacers 1–34), and lineage IV by the signature spoligotype of the EA-I family. The remaining strain families were confined to lineage subbranches, including the Haarlem family, confined to SSTs 4, 5, and 6 of lineage II, and the Delhi family, confined to lineage III, almost exclusively within SSTs 11, 12, 13, 26, and 27. The relationship between SST, lineage, spoligotype pattern, and IS*6110* RFLP pattern are shown in more detail in Figure 3.

Seventy-one isolates shared identical RFLP and spoligotype patterns with one or more isolate, grouped in 23 clusters, of which 10 possessed RFLP patterns containing five or fewer IS*6110* copies. Cluster sizes ranged from two to seven isolates, with a median cluster size of two. Isolate clusters were present in all lineages, but isolates with low copy number were confined to lineage II and IV. Three of the low copy number clusters, each characterized by a single IS*6110* band and distinct spoligotype pattern, were subdivided by SST, whereas among high copy number clusters, all isolates within a cluster possessed the same SST. To prevent introducing a selection bias, no correction was made for strain transmission.

### Polymorphisms and Antimicrobial Drug Resistance to Lineage

Having demonstrated a robust phylogeny for *M. tuberculosis* on the basis of neutral genetic variation, we annotated the tree with the nsSNPs. Most nsSNPs were rare within the isolate collection examined, many of which were represented uniquely. However, a number of nsSNPs known to confer drug resistance occurred frequently, including *rpoB* ^C 1367 G^, *rpoB* ^C 1367 T^, *rpoB* ^C 1351 T^ (*32**,**33*), *katG* ^A 944 C^ (*34**,**35*), *rpsL* ^A 128 G^ (*36*), and *inhA* ^C–15 T^ promoter mutation (*35*). These polymorphisms were distributed throughout the phylogeny, which implies that they arose independently on many occasions, presumably in response to the positive selection imposed by antimicrobial drug use. In contrast, an intergenic SNP, *oxyR-ahpC* **^G^** ^-46^ **^A^** (*37*), associated with, but not proven to cause, isoniazid resistance, occurred exclusively in lineage III and was present in all isolates within the lineage, which implies that this SNP may have arisen under neutral selection.

Although mutations conferring drug resistance were present in all lineages, the proportion of resistant and susceptible isolates varied between lineages. When antimicrobial drug susceptibility data were used, lineage III was positively associated with isoniazid resistance (51/62, p = 0.002), Lineages I and III were positively associated with streptomycin resistance (12/20, p = 0.0004 and 24/62, p = 0.004 respectively), and lineage IV was positively associated with fully susceptible isolates (30/62, p = 0.002). Rifampicin resistance was not associated with any lineage.

Genotypic analysis of phenotypically resistant isolates showed that among isoniazid-resistant isolates, the resistance-conferring mutation *katG* ^A 944 C^ was positively associated with lineage III (odds ratio [OR] 2.44, p = 0.016) and the *inhA* ^C –15 T^ promoter mutation positively associated with lineage IV (OR 3.28, p = 0.006). Among streptomycin-resistant isolates, the resistance-conferring mutation *rpsL* ^A 128 G^ was positively associated with lineage I (OR 7.83, p = 0.012) and negatively associated with lineage II (OR 0.32, p = 0.036). No genotype-lineage association was identified for rifampicin resistance. Different lineages have significant differences in their antimicrobial drug susceptibility to certain antimicrobial agents, perhaps demonstrating the effect of genomic environment on the probability of mutational events conferring resistance to these antimicrobial drugs.

### Lineage to Country of Birth

Drug resistance in countries with low TB incidence has been associated with foreign-born migrants (*11*); 44% of TB cases in England and Wales occur in the indigenous population (*22*). Information about country of birth was available for 225 (71%) of the patients; they represented 45 countries. Foreign-born patients had a median residency of 4 years in the United Kingdom. There was no significant difference between patients infected with susceptible or drug-resistant *M. tuberculosis* with respect to patient country or continent of birth. However, highly significant associations existed between continent of birth and lineage (Table 4). Lineages I, II, and III were significantly associated with southeastern Asia, Europe, and the Indian subcontinent, respectively. Lineage IV, in contrast, was globally distributed but had a negative association with Europe. This finding provides strong evidence for geographic structuring in *M. tuberculosis* populations.

**Table 4:** Relationship between *Mycobacterium tuberculosis* lineage and continent of birth of patient^a^

Linage	n	Europe				Africa				Indian subcontinent				Southeast Asia			
		Eur	Not Eur	OR	p value	Afr	Not Afr	OR	p value	ISC	Not ISC	OR	p value	SEA	Not SEA	OR	p value
I	17	5	12	0.86	0.997	1	16	0.17	0.079	1	16	0.12	0.034	10	7	28.8	< 0.00001
II	118	59	59	6.4	< 0.00001	33	85	1.34	0.473	19	99	0.2	< 0.00001	4	114	0.21	0.006
III	50	3	47	0.1	0.00001	9	41	0.58	0.245	37	13	11.31	< 0.00001	0	50	0.0	0.009
IV	40	5	35	0.25	0.005	15	25	2.04	0.08	15	25	1.36	0.514	5	35	1.66	0.166
Total	225	72	153			58	167			72	153			19	206		

^a^OR, odds ratio; Eur, Europe; Afr, Africa; ISC, Indian subcontinent; SEA, southeast Asia.

## Discussion

Synonymous nucleotide polymorphisms reflect neutral genomic variation, which remains informative, even in genes that have recently experienced positive selection attributable to introducing antimicrobial agents. By sequencing widely at multiple gene loci around the chromosome in a population sample of *M. tuberculosis* isolates, selection bias is avoided and all neutral variation within the sequenced regions will be identified. The indexed variation is highly unlikely to arise by convergence, which provides a robust base for constructing a phylogenetic tree. Our data support the belief that *M. tuberculosis* is a strictly clonal organism, with no evidence of lateral gene transfer.

Individual sSNPs are confined to clonally related organisms and accumulated by subsequent generations. Each of the lineages defined here can be defined on the basis of single sSNPs, yet the resultant maximum parsimony tree provides a robust and unifying phylogeny for *M. tuberculosis*. The documented population diversity is relatively recent, demonstrated by SSTs that represent almost all of the phylogenetic nodes. In contrast, *M. tuberculosis* and *M. bovis* separated from a common ancestor more distantly, which reinforces the evidence that *M. tuberculosis* could not have arisen from *M. bovis*, as previously thought.

Although we have sequenced a small number of *M. tuberculosis* complex isolates, our data support the evolutionary scenario described by Brosch et al. (*13*). *M. microti* is a subdivision of the *M. bovis* lineage, diverging after the separation of *M. bovis* from its common ancestor with *M. tuberculosis. M. africanum* type I, which cannot be distinguished from *M. tuberculosis* on the basis of neutral variation in the genes sequenced in this study, can be distinguished from *M. tuberculosis* SST-1 by the presence of the TbD1 region. Taken together, the sequence data support divergence of *M. africanum* type I from a common ancestor with *M. tuberculosis* before the subsequent divergence of *M. microti* and *M. bovis*. Analyzing silent nucleotide polymorphisms in *gyrB,* which has been used to distinguish members of the *M. tuberculosis* complex (*38**,**39*), would provide further neutral sequence variation to support the evolutionary scenario.

*M. tuberculosis* isolates in England and Wales represent four clearly defined lineages. Although the isolates were all obtained from patients residing in the United Kingdom, the patients represented 45 countries of birth, from four continents. The population is not globally representative; for example, few patients originated from the Americas. Nevertheless, the strong geographic structuring of the *M. tuberculosis* population is striking. *M. tuberculosis* is an obligate human pathogen, with a delay between initial infection and the development of clinical disease (often up to 5 years) and long periods of latency between disease control and subsequent clinical reactivation. The evolution and global dissemination of *M. tuberculosis* are by definition associated with the activities of its human host. Although foreign-born patients may have been infected with *M. tuberculosis* in the United Kingdom, the short median residency in the United Kingdom and the lack of strain clustering support the hypothesis that these are imported strains reflecting *M. tuberculosis* populations in the patient's country of birth. Clonal expansion of geographically restricted, genetically distinct lineages presumably reflects the previously geographically limited human population movements, with higher rates of transmission within, rather than between, geographic regions. No single *M. tuberculosis* lineage dominates in African-born patients. As in human populations, Africa appears to be a melting pot for genetic diversity. This fact may reflect the dissemination of *M. tuberculosis* by ancient human migration and trade routes but could be further elucidated by analysis of unselected isolates obtained in Africa.

Unlike lineages I, II and III, lineage IV is globally distributed, with no discernible geographic association. Not only is it the only lineage possessing the TbD1 region of difference, in common with *M. bovis*, *M. microti*, and *M. africanum* type I (*13*), but a large proportion of the isolates possess only a single IS*6110* copy, and isolates from the lineage are negatively associated with antimicrobial drug resistance. These data suggest that *M. tuberculosis* isolates from lineage IV are more closely related to the common ancestor of the *M. tuberculosis* complex, unexposed to antimicrobial selection pressure, and provide evidence to support the hypothesis that *M. tuberculosis* isolates possessing a single IS*6110* copy may be ancestral (*40**,**41*). In contrast, isolates possessing a high number of IS*6110* copies are present in all four *M. tuberculosis* lineages, which reflects independent IS*6110* transposition events in different parts of the phylogeny.

Geographic structuring of a clonal population will result in genetically and phenotypically distinct *M. tuberculosis* populations, which may explain, in part, the geographically variable response to vaccination with *M. bovis* BCG, or striking differences in clinical features, such as the predominance of extrapulmonary disease in patients originally from the Indian subcontinent. This finding may also have implications for the successful development of new TB vaccines. Nucleotide substitutions arising under neutral, positive, and negative pressure will all become fixed, inherited by all clonal descendants. Analyzing mutations that confer antimicrobial drug resistance provides an insight into this evolutionary process. By definition, resistance-conferring mutations are associated with phenotypic resistance absolutely. The genes involved all encode essential metabolic functions, restricting nonsynonymous nucleotide substitutions. The data demonstrate that the most frequently reported resistance-conferring mutations are present in all lineages, which implies that they have arisen independently on multiple separate occasions; however, phenotypically antimicrobial drug resistance is significantly associated with lineage. The significantly greater proportion of phenotypically resistant isolates with the *katG* ^315^ mutation in lineage III and the observation that the mutation is not present in all isolates within clonally related subbranches of the tree confirms the relatively recent influence of antimicrobial drug selection pressure. This finding implies that isolates within the lineage may be biochemically more susceptible to acquiring the same resistance conferring mutation. Rifampicin resistance and multidrug-resistant TB isolates were unrelated to lineage, although the numbers were relatively small (52 phenotypically rifampicin-resistant isolates, of which 46 were multidrug resistant).

We have shown that the sequenced *M. tuberculosis* strains, CDC1551 and H37Rv, are closely related and come from the same major lineage (lineage II, SST-2). This relation has implications for sSNP analyses based on comparison of only these genomes, collapsing subbranches and skewing any resultant phylogenetic tree (*14**,**29*). Although this tendency can be reduced slightly by comparing genomes that are genetically more divergent, the lack of horizontal gene transfer in a clonal population means that variation in other branches of the phylogeny will not be revealed. In fact, only four of the sSNPs described here can be resolved by genome comparison of the four completed genome sequences *M. tuberculosis* H37Rv (*17*), CDC1551 (*15*), strain 210 (www.tigr.org), and *M. bovis* (*18*). None were used in the SNP analysis performed by Gutacker et al. (*14*). By sequencing widely at multiple gene loci around the chromosome, we have identified all the indexable genetically neutral variation (sSNPs) within the sequenced regions. Although Sreevatsan et al. used a similar approach in their study of 26 structural genes, which included regions of all seven genes sequenced in this study (*19*), the isolates were selected from a large collection of *M. tuberculosis* strains in part on diversity in IS*6110* RFLP (introducing a bias towards high copy number strains), and the number of isolates sequenced at each locus varied, with no defined minimum dataset. We identified a similar level of genomic sequence diversity, but by using an unbiased population approach, we have shown phylogenetically significant neutral variation.

The phylogeny described is unambiguous and can be defined with a limited number of sSNPs. These could easily be identified with rapid screening techniques. Simultaneous identification of nsSNPs associated with antimicrobial drug resistance would provide data valuable for clinical, epidemiologic, and evolutionary purposes in a single, cost-effective, and highly portable format that is amenable to electronic database comparisons (*16*).
